# Risk of QT prolongation and torsade de pointes associated with exposure to hydroxyzine: re‐evaluation of an established drug

**DOI:** 10.1002/prp2.309

**Published:** 2017-04-21

**Authors:** Anne‐Françoise Schlit, Annie Delaunois, Aurore Colomar, Branderley Claudio, Luca Cariolato, Rossen Boev, Jean‐Pierre Valentin, Christopher Peters, Victor S. Sloan, Jürgen W. G. Bentz

**Affiliations:** ^1^ UCB PharmaBrusselsBelgium; ^2^ UBCGenevaSwitzerland; ^3^ UCB PharmaBulleSwitzerland; ^4^ UCB PharmaRaleighNorth CarolinaUSA; ^5^ Present address: Aurore Colomar, Université de MonsMonsBelgium

**Keywords:** Hydroxyzine, minimization, QT prolongation, risk, torsade de pointes

## Abstract

Several noncardiac drugs have been linked to cardiac safety concerns, highlighting the importance of post‐marketing surveillance and continued evaluation of the benefit‐risk of long‐established drugs. Here, we examine the risk of QT prolongation and/or torsade de pointes (TdP) associated with the use of hydroxyzine, a first generation sedating antihistamine. We have used a combined methodological approach to re‐evaluate the cardiac safety profile of hydroxyzine, including: (1) a full review of the sponsor pharmacovigilance safety database to examine real‐world data on the risk of QT prolongation and/or TdP associated with hydroxyzine use and (2) nonclinical electrophysiological studies to examine concentration‐dependent effects of hydroxyzine on a range of human cardiac ion channels. Based on a review of pharmacovigilance data between 14th December 1955 and 1st August 2016, we identified 59 reports of QT prolongation and/or TdP potentially linked to hydroxyzine use. Aside from intentional overdose, all cases involved underlying medical conditions or concomitant medications that constituted at least 1 additional risk factor for such events. The combination of cardiovascular disorders plus concomitant treatment of drugs known to induce arrhythmia was identified as the greatest combined risk factor. Parallel patch‐clamp studies demonstrated hydroxyzine concentration‐dependent inhibition of several human cardiac ion channels, including the ether‐a‐go‐go‐related gene (hERG) potassium ion channels. Results from this analysis support the listing of hydroxyzine as a drug with “conditional risk of TdP” and are in line with recommendations to limit hydroxyzine use in patients with known underlying risk factors for QT prolongation and/or TdP.

**Abbreviations**
CSI: cardiac safety index
DDD: defined daily dose
ECG: electrocardiogram
HCP: healthcare professional
hERG: ether‐a‐go‐go related gene
IBD: International Birth Date
ICH: International Conference on Harmonization
MedDRA: Medical Dictionary for Regulatory Activities
PTs: preferred terms
SMQ: standard MedDRA query
TTO: time to onset
WHO: World Health Organization

## Introduction

A growing number of noncardiac drugs have been found to delay cardiac repolarization, causing QT interval prolongation^1^ and predisposing patients to an increased risk of potentially fatal ventricular arrhythmias, known as torsade de pointes (TdP) (Letsas et al. 2007; Woosley et al. 2016; Sanguinetti and Tristani‐Firouzi 2006; Yap and Camm 2003). In several cases, the cardiac events were associated with exceeding the recommended dose or with pharmacokinetic interactions with concomitant drugs known to inhibit cytochrome P450 isoenzymes and decrease metabolism of the antihistamines (Yap and Camm 2003). In some cases, patients had an elevated baseline risk of QT prolongation, for example due to concomitant treatment with medications that potentially prolonged the QT interval (e.g., antiarrhythmics, antipsychotics), or the presence of underlying heart conditions (Yap and Camm 2003; Kannankeril et al. 2010). These drugs include the second‐generation, non‐sedating antihistamines astemizole and terfenadine, withdrawn from the market in Europe and the United States (in 1997 and 1999, respectively) due to cardiac safety concerns (Kannankeril et al. 2010; World Health Organization, 2011; Davila et al. 2006).

In past years, concerns have been raised regarding the cardiac safety of hydroxyzine, a first generation sedating antihistamine used for the management of anxiety and pruritus (Atarax Summary of Product Characteristics, 2016). Hydroxyzine is currently authorized in 33 countries worldwide, with marketing authorization granted in 1955. This was before the adoption of International Conference on Harmonization (ICH) guidelines to assess the risk of QT prolongation as a result of drug treatment. The ICH clinical (E14) (ICH Harmonised Tripartite Guideline E14, 2005) and non‐clinical (S7B) (ICH Harmonised Tripartite Guideline S7B, 2005) guidelines were introduced in response to regulatory concerns over a growing number of reports of QT prolongation associated with non‐antiarrhythmic drugs. For long‐established drugs, such as hydroxyzine, post‐marketing surveillance and continued research plays an essential role in assessing the benefit‐risk profile of the drug in clinical practice, and identifying any potential safety concerns including QT prolongation and TdP.

Prior to this analysis, post‐marketing evidence of hydroxyzine‐mediated QT prolongation and TdP was limited to a few case reports (Acosta‐Materan et al. 2016; Sakaguchi et al. 2008; Vigne et al. 2015). Data from nonclinical studies have demonstrated an inhibitory effect of hydroxyzine on human cardiac ether‐a‐go‐go‐related gene (hERG) potassium ion channels, with IC_50_ values ranging from 0.62 *μ*mol/L at 37°C to 0.16 *μ*mol/L at room temperature (Sakaguchi et al. 2008; Lee et al. 2011). Inhibition of hERG channels can delay action potential repolarization (due to a reduction in the outward potassium current peak) and potentially cause QT interval prolongation and the associated cardiac risks, although there are limitations to linking this data to real‐world safety concerns (Letsas et al. 2007; Kannankeril et al. 2010; Redfern et al. 2003; Valentin et al. 2010; Gintant et al. 2016).

As marketing authorization holder of the originator product, we have conducted an independent, thorough and comprehensive review of hydroxyzine safety data to re‐evaluate the cardiac safety of this long‐established antihistamine. Here we report a summary of all spontaneously reported adverse events associated with QT prolongation and/or TdP in patients administered hydroxyzine. Our results are described according to hydroxyzine dosage, underlying medical conditions, and potential cardiac risk factors. New non‐clinical data are also presented that examine the dose‐dependent effects of hydroxyzine on multiple cardiac ion channels, including the potassium ion channel responsible for rapid cardiac repolarization (hERG).

## Materials and Methods

### Review of the sponsor pharmacovigilance database

The sponsor pharmacovigilance database provides spontaneous adverse event reports from worldwide sources (including healthcare professionals [HCPs], consumers, non‐interventional studies, and the literature) for all sponsor marketed products, as well as serious adverse event reports from clinical trials of any sponsor compounds in development. For this study we conducted a review of all adverse event reports occurring between 14th December 1955 (the International Birth Date [IBD] for hydroxyzine) and 1st August 2016, for formulations containing hydroxyzine as the active compound.

In brief, the sponsor database was searched for hydroxyzine and Medical Dictionary for Regulatory Activities (MedDRA) preferred terms (PTs) for TdP and QT prolongation. Six narrow standard MedDRA query (SMQ) PTs, considered highly likely to represent adverse events associated with QT prolongation and/or TdP, were used: electrocardiogram (ECG) QT prolonged, ECG QT interval abnormal, long QT syndrome, long QT syndrome congenital, TdP, and ventricular tachycardia. An additional 14 broad SMQ PTs were used to identify all possible related cases: cardiac arrest, cardiac death, cardiac fibrillation, cardio‐respiratory arrest, ECG U‐wave abnormality, ECG repolarization abnormality, loss of consciousness, sudden cardiac death, sudden death, syncope, ventricular arrhythmia, ventricular fibrillation, ventricular flutter, and ventricular tachyarrhythmia. MedDRA version 19.1 was used for the coding of adverse events.

Information on gender, age, year of incident, dose, indication, treatment time, concomitant drugs, and known risk factors was collected from each case report, when available. A reference list of concomitant drugs associated with QT prolongation and/or onset of TdP was retrieved from CredibleMeds^®^ (crediblemeds.org, accessed on 12th September 2016) (Woosley et al. 2016). Underlying medical conditions and known risk factors were identified based on the published literature (see Table 1).

**Table 1:** Potential risk factors for QT interval prolongation identified in the literature (Zeltser et al. 2003)

Cardiovascular disorders	Coronary artery disease
	Heart failure
	Ventricular tachyarrhythmias
	Dilated cardiomyopathy
	Hypertrophic cardiomyopathy
	Left ventricular hypertrophy
	Baseline QT interval prolongation
	Hypertension
	Bradycardia (SA nodal dysfunction, AV block)
	Myocarditis
Metabolic abnormalities	Hypokalemia
	Hypocalcemia
	Hypomagnesemia
Endocrine disorder	Hypothyroidism
	Hyperparathyroidism
	Pheochromocytoma
	Hyperaldosteronism
Intracranial pathology	Subarachnoid hemorrhage
	Cerebrovascular accident
	Head injury
	Encephalitis
Liver disease	Cirrhosis
	Hepatic failure
Renal disease	
Diabetes mellitus	
Anorexia nervosa/starvation	
Bulimia	
Obesity	
Liquid protein diet	
Human immunodeficiency virus (HIV) infection	
Older age	
Female gender	
Lupus erythematosus (Cardoso et al. 2005)	
Polypharmacy (Khan 2006)	
Hypothermia	
Cytochrome P450 isoenzyme CYP3A4 inhibitors	
Ion channel mutations/polymorphisms (hERG)	

This table has been retrieved from Camm et al. (2004) and updated with other references.

AV, atrioventricular; hERG, human ether‐a‐go‐go‐related gene; QT, QT interval is defined as the time between the beginning of the Q wave and the end of the T wave of the PQRST cardiac activity cycle; SA, sinoatrial.

In order to evaluate causality, the maximum time to onset (TTO) was set at 7 days to include all cases potentially linked to hydroxyzine intake. In summary, the cases were assessed using the following strategy: TTO (<7 days); daily dose (mg); presence of risk factors/underlying medical conditions; and positive dechallenge/rechallenge.

### Exposure data

Exposure data for hydroxyzine was estimated based on sales data from 1st January 2007 to 31st July 2016 (these data were not available prior to 2007). The defined daily dose (DDD) was 75 mg, in accordance with the World Health Organization (WHO), and patient‐years of exposure was calculated using the following formula:

$$\text{Patient}-\text{years}=\frac{\text{Total mg of product distributed}/\text{DDD}}{\text{365.25 days in year}}$$

### Nonclinical electrophysiological studies

In vitro whole‐cell patch‐clamp experiments were performed using human embryonic kidney 293 (HEK293) cells to assess the concentration‐dependent effects of hydroxyzine on the peak amplitude of tail currents mediated by stably expressed hERG potassium channels. A conventional manual patch‐clamp assay technique was used (Hamill et al. 1981). Increasing concentrations of hydroxyzine (0.01, 0.03, 0.1, 0.3, and 3 *μ*mol/L), all within the clinically relevant range, were tested at room temperature (22 ± 2°C) and at near‐physiological temperature (35 ± 1°C).

In a second nonclinical study, patch‐clamp techniques (Hamill et al. 1981) were used to assess the concentration‐dependent effects of hydroxyzine on seven different human cardiac ion channels (voltage‐gated sodium channel, Nav1.5; voltage‐gated potassium channels, Kv4.3, Kv1.5 and KCNQ1/MinK; voltage‐gated calcium channel, Cav1.2; inward‐rectifier potassium channel, Kir2.1; and hyperpolarization‐activated cyclic nucleotide‐gated channel, HCN4). The channels were expressed in different cell lines: Chinese hamster ovary (CHO) cells for Cav1.2, Kv1.5 and KCNQ1/MinK, or HEK293 cells for the other channels. The concentrations of hydroxyzine (0.01, 0.03, 0.3, 3, 30 *μ*mol/L), tested at room temperature, covered and exceeded the estimated therapeutic exposure range.

Further methodological details are provided in supplemental materials. All experiments were compliant with ICH S7B guidelines for the nonclinical evaluation of the potential for QT prolongation (ICH Harmonised Tripartite Guideline S7B, 2005).

### Statistical analysis

For nonclinical data, the IC_50_ was fitted with the mono‐exponential equation: y = *V*_max_ × (X^*n*^) / [(k^n^) + (X^*n*^)] where y denotes percentage inhibition of the current of interest at hydroxyzine concentration X; *V*_max_, k and *n* correspond to the maximal current inhibition of the hERG tail current (data fixed to 100), IC_50_ and Hill coefficient, respectively.

The cardiac safety index (CSI) of hydroxyzine was calculated by dividing the estimated IC_50_ value for inhibition of the hERG tail current by the C_max,free_ of hydroxyzine at a dose of 50 mg (0.01 *μ*mol/L). CSI evaluation at hydroxyzine doses >50 mg remains theoretical and incomplete, as pharmacokinetics for higher concentrations of hydroxyzine in humans are unknown.

## Results

### Pharmacovigilance case reports of QT prolongation and/or TdP

Based on marketing experience between 14th December 1955 and 1st August 2016, a total of 59 case reports of QT prolongation and/or TdP were retrieved from the safety database using the narrow SMQ PTs for QT prolongation/TdP (Table 2). These cases represent spontaneous reports of adverse events from HCPs, consumers and the literature. Using sales data (available since 2007) the total exposure to hydroxyzine was estimated to be 8,913,469 patient‐years, with 34 case reports of QT prolongation and/or TdP over this period and an average reporting rate of 3.81 cases per 1,000,000 patient‐years.

**Table 2:** Characteristics of the narrow SMQ QT prolongation/TdP cases

Characteristic	TdP/QT prolongation cases (*n* = 59)
Age, *n* (%)	
Below 18 years	1 (1.5)
18 to <65 years	41 (69.5)
≥65 to <81 years	15 (25.5)
Unknown	2 (3.5)
Gender, *n* (%)	
Male	18 (31)
Female	39 (66)
Unknown	2 (3)
Report type, *n* (%)	
Spontaneous reports	11 (19)
Spontaneous literature	8 (13.5)
Spontaneous regulatory authority	39 (66)
Study–UCB Non‐interventional study	1 (1.5)
Report Source, *n* (%)	
Healthcare professional	58 (98.5)
Consumer	1 (1.5)
Indications, *n* (%)	
Pre‐medication	9 (15.5)
Voluntary intoxication/suicide attempt	12 (20.5)
Pruritus/Urticaria	6 (10)
Anxiety	5 (8.5)
Depression	4 (7)
Bladder disorder	1 (1.5)
Drug hypersensitivity	1 (1.5)
Insomnia	1 (1.5)
Migraine	1 (1.5)
Unknown	19 (32.5)
Time to onset (TTO) of event	
Median (range)	1 day (few minutes–16 months)
Cases with known TTO, *n* (%)	38 (64)
TTO (excluding cases of overdose)	
Median (range)	3 days (<1 day–16 months)
Cases with known TTO, *n* (%)	23 (39)
Outcome, *n* (%)	
Fatal	8 (14)
Not recovered	6 (10)
Recovered with sequelae	2 (3)
Recovered	27 (46)
Unknown	16 (27)
Dechallenge,^a^ *n* (%)	
Positive	16 (27)
Negative	2 (3)
Rechallenge, *n* (%)	
Positive	0
Negative	0

*n* refers to the number of cases.

QT, QT interval is defined as the time between the beginning of the Q wave and the end of the T wave of the PQRST cardiac activity cycle; SMQ, standard MedDRA query; TdP, torsade de pointes; TTO, time to onset.

a For the remaining cases, dechallenge was not reported, or reported as unknown.

QT prolongation and/or TdP events were reported in patients across all indications, with a median TTO (specified in 64% of cases) of 1 day following hydroxyzine administration. Among cases that described the TTO, 15 were reported following an overdose of hydroxyzine and patients experienced adverse events within 1 day. Excluding these 15 reports of overdose, the median TTO was 3 days. Overall, 14 out of the 59 cases (excluding cases with overdose) had a TTO of <7 days; considered a sufficiently long period to capture all cases potentially linked to hydroxyzine intake. Of these cases, the most reported risk factor (50% of cases) was the combination of cardiovascular disorders with concomitant administration of drugs known to induce QT prolongation/TdP. Positive dechallenge was reported in 16/59 cases, with the adverse event subsiding or stopping upon discontinuation of drug therapy. In the majority of cases multiple medications were discontinued, including 12 cases in which other drugs linked to QT interval prolongation and/or TdP had also been discontinued.

Among the 59 case reports, a total of 69 events were identified (individual patients could experience >1 event). This included 41 events of ECG QT prolonged, 18 events of TdP, and 10 events of ventricular tachycardia (Table 3). There were no events of ECG abnormal QT interval, long QT syndrome or congenital long QT syndrome. QT prolongation and/or TdP were reported across the entire range of hydroxyzine doses used; 27 patients were exposed to hydroxyzine ≤100 mg, 18 patients were exposed to a daily dosage >100 mg, and in 14 cases the dosage of hydroxyzine was unknown. In total, 8 fatalities were identified across dosage groups in the narrow SMQ search, 7 of which had underlying risk factors. The remaining fatality was associated with an intentional overdose of hydroxyzine, possibly in combination with other medications, in a patient with missing information on cardiovascular medical history and concomitant medications (Table 4).

**Table 3:** Events of interest from the narrow SMQ search, stratified according to dosage range of hydroxyzine with a separate group for events with pre‐medication indication

Hydroxyzine dose	Events of interest			Total number of events/Total number of cases
	Ventricular tachycardia	ECG QT prolonged	Torsade de Pointes	
≤100 mg/day	2	14	7	23/19
100 to ≤300 mg/day	—	4	—	4/4
>300 mg/day	—	10	4	14/13
Unknown	4	9	3	16/14
Pre‐medication 25–400 mg^a^	4	4	4	12/9
Number of events	10	41	18	69/59

ECG, electrocardiogram; QT, QT interval is defined as the time between the beginning of the Q wave and the end of the T wave of the PQRST cardiac activity cycle; SMQ, standard MedDRA query.

a Pre‐medication, a single dose of hydroxyzine before surgery; data were pooled across different pre‐medication hydroxyzine doses.

**Table 4:** Summary of fatalities from the narrow SMQ search (*n* = 8)

Preferred term of interest	Risk factors identified			Causality of death in relation to hydroxyzine administration
	Dosage (mg/day)	Relevant medical history	Relevant concomitant drugs	
ECG QT prolonged	100–300	Yes (Cardiovascular disorders)	Yes	Information not provided
ECG QT prolonged	≤100	Yes (Cardiovascular disorders)	Yes	Information not provided
ECG QT prolonged	≤100	Yes (Renal disorders)	Yes	Information not provided
TdP	Unknown	Yes (HIV infection)	Yes	Considered unrelated
TdP	≤100	Yes (Cardiovascular disorders)	Yes	Information not provided
TdP	>300	Unknown	Unknown	Intentional overdose^a^
Ventricular tachycardia	Unknown	Yes (Underlying lupus erythematosus)	Yes	Considered unrelated
Ventricular tachycardia	Unknown	No	Yes	Information not provided

ECG, electrocardiogram; *n*, refers to the number of cases; QT, QT interval is defined as the time between the beginning of the Q wave and the end of the T wave of the PQRST cardiac activity cycle; SMQ, standard MedDRA query; TdP, torsade de pointes.

a Information relating to the overdose is limited and the intake of multiple medications cannot be excluded.

### Underlying risk factors for QT prolongation and/or TdP

All 27 case reports of QT prolongation and/or TdP in patients exposed to hydroxyzine ≤100 mg (maximum recommended dosage) had at least one underlying independent risk factor for the onset of the QT prolongation/TdP (Table 5). The most common risk factor was the combination of cardiovascular disorders with administration of concomitant drugs known to induce QT prolongation/TdP (48.5% of cases). Use of concomitant drugs known to induce QT prolongation/TdP in the absence of other known risk factors was associated with 18.5% of cases. Fifteen cases specified the TTO with a median of 3 days.

**Table 5:** Most reported risk factors among the 27 cases exposed to hydroxyzine <100 mg

Risk factors of acquired long QT syndrome	Cases, *n* (%)
Cardiovascular disorders + concomitant drugs known to induce QT prolongation/TdP	13 (48.5)
Concomitant Drugs known to induce QT prolongation/TdP	5 (18.5)
Cardiovascular disorders	2 (7.5)
Metabolic abnormalities + concomitant drugs known to induce QT prolongation/TdP	2 (7.5)
Genetic disorders	2 (7.5)
Metabolic abnormalities	1 (3.5)
Renal disease	1 (3.5)
Renal disease + concomitant drugs known to induce QT prolongation/TdP	1 (3.5)
Total	27 (100)

*n*, refers to the number of cases; TdP, torsade de pointes; QT, QT interval is defined as the time between the beginning of the Q wave and the end of the T wave of the PQRST cardiac activity cycle.

Of the 59 case reports, 15 reports were in elderly patients (≥65 years of age) and 39 reports were in females (Tables S1, S2). The most common risk factor in both patient groups was underlying cardiovascular disorders in combination with the use of concomitant drugs known to induce QT prolongation/TdP; associated with 53.0% of case reports in elderly patients and 33.5% of case reports in female patients. Concomitant use of drugs known to induce QT prolongation/TdP (in the absence of other known risk factors) accounted for 20.0% and 15.5% of case reports in elderly and female patients, respectively. Among cases with a known TTO, the median TTO was 2 days in elderly patients and 1 day in female patients. Of note, both patient groups included cases of overdose that reduced the median TTO. Excluding these cases, the median TTO was 3 days for both groups.

From the broad SMQ search, 226 cases were retrieved; excluding the 59 cases identified using the narrow SMQ PTs, 167 cases were reported. Of these, 24 cases were reported with a TTO <7 days, including 13 reports of loss of consciousness and syncope (symptoms that may describe conditions associated with QT prolongation and TdP), 10 cardiac and/or cardiorespiratory arrests (including four fatal cases), and one case of ECG repolarization abnormality. This latter case was particularly supportive for our analysis because it described a suggestive event for the development of QT prolongation/TdP, and also reported concomitant medications known to induce QT prolongation and TdP.

### Concentration‐dependence of cardiac ion channel inhibition

**Figure 1:**
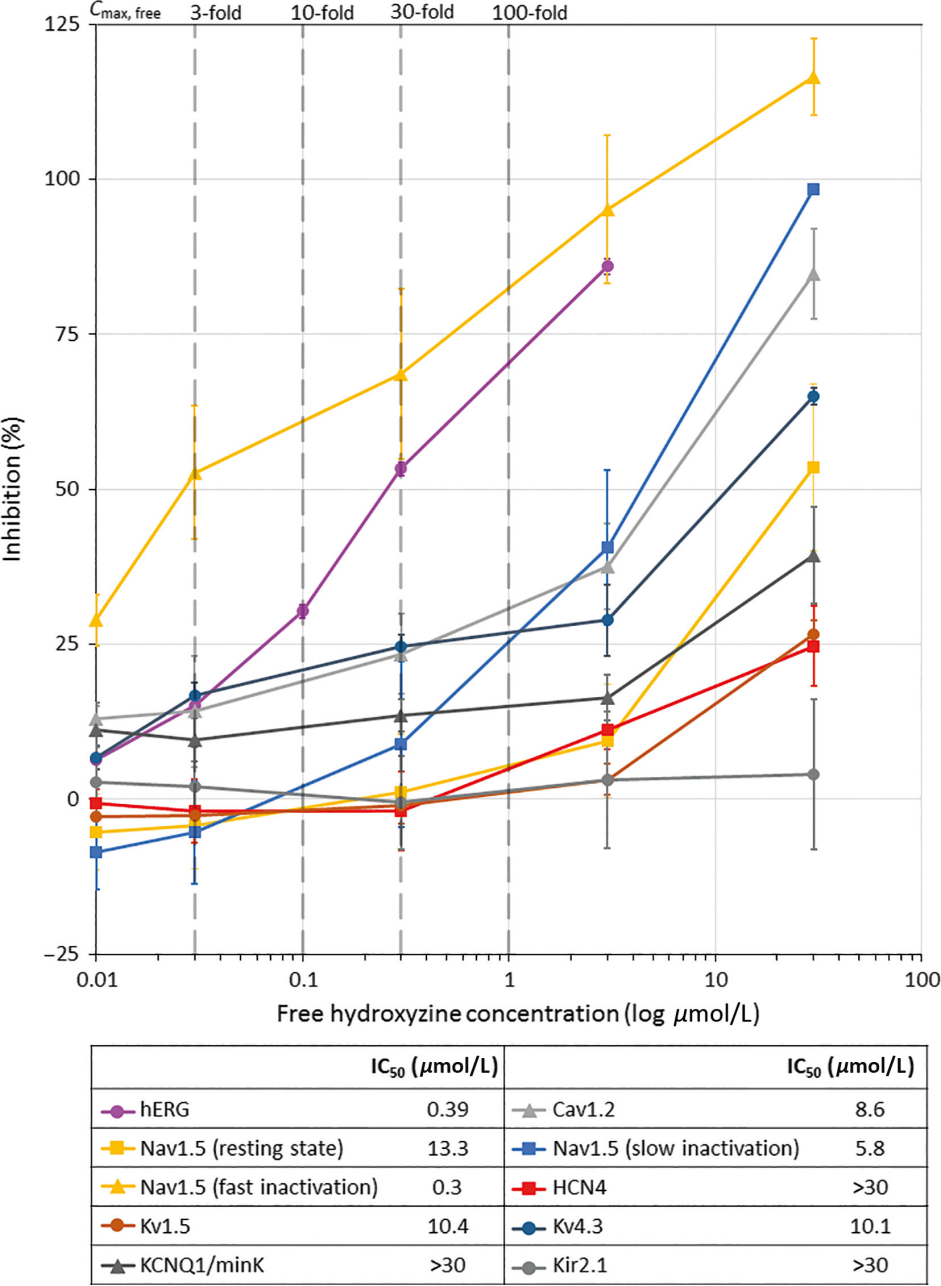
Hydroxyzine concentration‐response curves for the inhibition of different cardiac ion channels in vitro. Cav, voltage‐gated calcium channel; HCN, hyperpolarization‐activated cyclic nucleotidegated channel; hERG, human ether‐a‐go‐go‐related gene; IC_50_, half maximal inhibitory concentration; Nav, voltage‐gated sodium channel; KCNQ1, voltage‐gated potassium channel subfamily Q member 1; Kir, inward‐rectifier potassium channel; Kv, voltage‐gated potassium channel; minK, minimal potassium channel subunit.

In vitro patch‐clamp studies examined the effects of clinically‐relevant hydroxyzine concentrations on a range of ion channels that influence cardiac action potential repolarization and QT interval. Hydroxyzine was found to block peak hERG channel tail currents in a concentration‐dependent manner, with an IC_50_ of 0.39 *μ*mol/L at near‐physiological temperature (*n* = 4; Fig. 1). Inhibitory effects of hydroxyzine were also observed for specific voltage‐gated sodium and calcium cardiac ion channels (Nav1.5 and Cav1.2; Fig. 1). These findings demonstrated potential QT prolongation effects of hydroxyzine, via inhibition of cardiac hERG channels that may be compensated for by inhibition of other cardiac ion channels.

## Discussion

Hydroxyzine was launched in 1955, when there was no information on the molecular mechanisms underlying QT prolongation and no regulatory guidelines for assessing the potential cardiac risk of a new drug. The hERG channel was first described by Warmke and Ganetzky (1994) and the following year Sanguinetti et al. (1995) demonstrated a link between the channel and inherited or acquired (i.e. drug‐induced) prolongations of the QT interval. In 2005, the ICH introduced clinical guidelines for assessing the risk of QT prolongation in drugs (ICH Harmonised Tripartite Guideline S7B, 2005; ICH Harmonised Tripartite Guideline E14, 2005). It is only in recent years that non‐clinical studies and post‐marketing safety analyses have suggested a potential increased risk of QT interval prolongation and/or TdP associated with exposure to hydroxyzine (Acosta‐Materan et al. 2016; Redfern et al. 2003; Valentin et al. 2010).

In this study we have re‐evaluated the cardiac safety profile of hydroxyzine using a combined methodological approach. A cumulative review of case reports from the sponsor pharmacovigilance safety database, using a narrow SMQ for QT prolongation and/or TdP, identified 59 case reports potentially linked to hydroxyzine treatment up to 1st August 2016, with an average reporting rate of 3.81 QT prolongation and/or TdP events/1,000,000 patient‐years of exposure (based on data since 2007).

All of the 59 cases reported involved either underlying medical conditions or concomitant medications that constituted at least one baseline risk factor (and often multiple risk factors) for such events, or were associated with an intentional overdose. Potential risk factors included a history of cardiac disorders (such as bradycardia, congestive heart failure, or cardiomyopathies), metabolic abnormalities, genetic disorders, renal disease, and concomitant medications associated with QT prolongation and/or TdP.

The results presented here are aligned with the listing of hydroxyzine as having a “conditional risk of TdP”; with QT prolongation and/or TdP only observed in patients with underlying risk factors or following intentional overdose (Woosley et al. 2016). Findings suggest that the risk did not differ across indications; although, in a number of cases the indication was not recorded. No clear dose‐dependent effects of hydroxyzine were observed for the case reports, based on the numerical distribution of cases across the range of hydroxyzine dosages. However, all 15 narrow SMQ case reports describing QT prolongation and/or TdP in patients with a hydroxyzine dose >300 mg/day concerned suicide attempts that included the intake of hydroxyzine. Across different dose, age, and gender groups the most frequently reported risk factor was the combination of cardiovascular disorders with administration of concomitant drugs that are known to contribute to the development of QT prolongation and/or TdP. Other factors, including pharmacokinetic interactions and overdose, can contribute to the onset of TdP (Yap and Camm 2003; Kannankeril et al. 2010) and accounted for some of the cases reported here.

A median TTO of 3 days (excluding cases of overdose) was observed among different dose, age and gender groups indicating a possible causal relationship between hydroxyzine administration and the event of interest, and is similar to the TTO observed in recently published case reports (Sakaguchi et al. 2008). Analogous findings were observed among the broad SMQ cases reporting a suggestive TTO. However, it is important to highlight that the broad SMQ PTs may describe conditions other than QT prolongation and TdP.

Taken together, these findings indicate a need to adhere to the recent recommendation that hydroxyzine should be avoided in patients who have underlying risk factors for heart rhythm disturbances, or who are taking concomitant medicines that may increase the risk of QT prolongation (European Medicines Agency, 2015). Reports from the literature of other drugs with a “conditional risk of TdP” suggest that several factors can contribute to the risk of QT prolongation and TdP (Letsas 2010; Zeltser et al. 2003). These findings can be explained by the concept of a repolarization reserve, which typically requires multiple risk factors acting conjointly to become exhausted, permitting the occurrence of cardiac electrophysiological disturbances (Kannankeril et al. 2010; Roden 1998, 2008). The influence of any individual factor that may contribute to cardiac action potential hyperpolarization is expected to vary from patient to patient and is dependent on the existence and intensity of other underlying risk factors for QT prolongation and/or TdP. Our results suggest that in patients with no other risk factors, the likelihood of adverse cardiac events is very low when hydroxyzine is taken at the recommended dose. In many of the 59 cases reported here, multiple other risk factors were identified, or the cardiac events were associated with intentional overdose, and any specific contribution of hydroxyzine intake is unknown.

Since the CSI decreases with higher *C*_max,free_ values, the risk of arrhythmia is likely to be increased in patients with high plasma concentrations of hydroxyzine, such as those receiving high dosages (>100 mg/day), the elderly (who may have a decreased clearance), and those exposed to interactions of hydroxyzine with concomitant drugs. In line with previous reports that hydroxyzine can inhibit hERG potassium channels with IC_50_ values ranging from 0.62 *μ*mol/L down to 0.16 *μ*mol/L, (Sakaguchi et al. 2008; Lee et al. 2011) we report an estimated IC_50_ of 0.39 *μ*mol/L for hydroxyzine at near‐physiological temperature, when tested over a clinically‐relevant concentration range. This IC_50_ value generated a CSI value of 39 for a 50 mg dose of hydroxyzine (ratio between hERG IC_50_ and *C*_max,free_ of hydroxyzine [0.01 *μ*mol/L]), assuming no other risk factors. This is above the recommended CSI ratio of 30 and suggests that hydroxyzine has an acceptable safety margin for proarrhythmia risk (Redfern et al. 2003). Results presented here also demonstrate inhibitory effects of hydroxyzine on specific sodium and/or calcium cardiac ion channels. Previous studies suggest that inhibitory effects on these channels may compensate for any hERG channel blockade and protect against TdP or other “hERG‐related” arrhythmias (Kramer et al. 2013; Milberg et al. 2005; Oros et al. 2010; Orth et al. 2006; Saint 2008; Zygmunt et al. 2011).

Limitations of this study include the use of a post‐marketing database, which may contain incomplete reports, and the well‐described under‐reporting in these databases. Although measures were taken to perform appropriate control experiments and manage experimental conditions for the patch‐clamp studies, (Gintant et al. 2006; Hancox et al. 2008) there are limitations to the extent to which nonclinical data can be linked to real‐world safety data (Valentin et al. 2010). Furthermore, blockade of hERG channels does not necessarily lead to clinically significant arrhythmias such as TdP, and not all drugs causing TdP are potent hERG blockers (Yang et al. 2001). There is also no fixed relationship between the extent of QT prolongation and the risk of TdP (Gintant 2008).

When taken at a maximum recommended dosage of 100 mg/day, hydroxyzine can provide important clinical benefits for many patients across indications. To ensure that the benefit‐risk ratio is positive for all patients receiving hydroxyzine, precautions should be taken to limit its use in those with known risk factors for QT prolongation and TdP regardless of patient age and the daily dose taken. This includes, but is not limited to, pre‐existing cardiac conditions, metabolic abnormalities, and/or exposure to concomitant medications associated with QT prolongation and/or TdP. The use of hydroxyzine is not recommended in the elderly due to a prolonged elimination half‐life, but if unavoidable, a maximum dose of 50 mg/day should be used.

In summary, based on an overall assessment of data from the literature, non‐clinical studies, and pharmacovigilance data we have re‐evaluated the risk of QT prolongation and/or TdP associated with exposure to hydroxyzine, a drug used in clinical practice since the 1950s. There are several implications of our findings. Firstly, they confirm that hydroxyzine can be listed as a drug with “conditional risk of TdP”. Secondly, they demonstrate that the combination of cardiovascular disorders with concomitant treatment of drugs known to induce arrhythmia is the greatest risk factor for the onset of QT prolongation/TdP after hydroxyzine administration. Lastly, they emphasize the importance of continual re‐evaluation of potential safety issues of older drugs using modern knowledge and standards. Taking into account the full critical evaluation of information presented here, and the implementation of appropriate measures to ensure that hydroxyzine use is carefully considered in those patients with an underlying risk of QT prolongation and/or TdP, the benefit‐risk balance of hydroxyzine remains positive for the recommended doses and indications of anxiety and pruritus.

## Author Contributions

Substantial contributions to study conception/design, or acquisition/analysis/interpretation of data: A‐FS, AD, AC, BC, LC, RB, J‐PV, CP, VSS, JB; Drafting of publication, or revising it critically for important intellectual content: A‐FS, AD, AC, BC, LC, RB, J‐PV, CP, VSS, JB; Final approval of the Publication: A‐FS, AD, AC, BC, LC, RB, J‐PV, CP, VSS, JB.

## Diclosure

A‐FS, AD, RB, J‐PV, VSS and JB are employees and shareholders of UCB Pharma; AC and CP are former employees of UCB Pharma; BC and LC are contract employees for UCB Pharma.

## Supporting information


**Supplemental Methodology.** Non‐clinical electrophysiological studies.
**Table S1.** Most reported risk factors among the 15 cases in elderly patients.
**Table S2.** Most reported risk factors among the 39 cases in female patients.

## Note

1 QT interval is defined as the time between the beginning of the Q wave and the end of the T wave of the PQRST cardiac activity cycle observed via an electrocardiogram (ECG).
