# Supplementary material for: Risk of QT prolongation and torsade de pointes associated with exposure to hydroxyzine: re‐evaluation of an established drug
Source: Pharmacol Res Perspect. 2017 Apr 21;5(3):e00309. doi: 10.1002/prp2.309 (PMC5415947; doi:10.1002/prp2.309)
Supplement: Supplementary file 1 — Supplemental Methodology. Non‐clinical electrophysiological studies. Table S1. Most reported risk factors among the 15 cases in elderly patients. Table S2. Most reported risk factors among the 39 cases in female patients. [file PRP2-5-e00309-s001.docx]

SUPPLEMENTAL MATERIALS

HEK293 cells stably expressing the hERG channel were cultured in Dulbecco’s Modified Eagle Medium (DMEM) supplemented with 10% (v/v) fetal bovine serum (FBS), 1.2 mg/mL Geneticin and 1% (v/v) penicillin/streptomycin, and maintained at 37°C in a 95% air/5% CO_2_ atmosphere.

For electrophysiological studies, the cells were seeded onto 35 mm culture dishes. Extracellular solution contained (in mmol/L): 137 NaCl, 4 KCl, 1.8 CaCl_2_, 1 MgCl_2_, 10 HEPES (N-[2-hydroxyethyl] piperazine-N’-[2-ethane sulfonic acid]), and 10 glucose, adjusted to a final pH of 7.40 ± 0.02 using NaOH. Intercellular (patch pipette) solution contained (in mmol/L): 130 KCl, 1 MgCl_2_, 5 EGTA (ethylene glycol-bis [2-aminoethylether]-N,N,N’,N’-tetraacetic acid), 10 HEPES, and 5 MgATP, adjusted to a final pH of 7.20 ± 0.02 using KOH. A conventional manual patch-clamp assay technique was used to monitor hERG tail current amplitude using the whole-cell configuration.^1^ Pipette resistance ranged from 3.4 to 5.6 MΩ. Cells were maintained at a holding potential of -80 mV, and hERG currents were activated by a 1 second depolarisation to +30 mV followed by 1 second repolarisation to ‑40 mV. This stimulation protocol was repeated each 20 seconds (frequency: 0.05 Hz). Control cells were treated with vehicle (extracellular solution). E-4031 (0.1 µM), a known inhibitor of the hERG-encoded potassium channel, was used as a positive control.

Seven other human cardiac ion channels were also tested. Cav1.2, Kv1.5 and KCNQ1/MinK were stably expressed in CHO cells and Nav1.5, Kv4.3, Kir2.1 and HCN4 channels were stably expressed in HEK293 cells. A whole-cell manual patch-clamp assay^1^ was used to assess the concentration-dependent effects of hydroxyzine. Cells were plated out onto glass cover slips and mounted on an inverted microscope and continuously bathed in control solution. Extracellular solution varied depending on the type of current being recorded and contained (in mmol/L): 140 TEACl, 2 BaCl_2_, 1 MgCl_2_, 10 HEPES, and 10 glucose, adjusted to pH 7.3 using TEAOH for Cav1.2 channels; 110 NaCl, 30 KCl, 1.8 CaCl_2_, 1 MgCl_2_, 10 HEPES, and 5 glucose, adjusted to pH 7.35 using NaOH for HCN4 channels; 46 NaCl, 91 TEACl, 4 KCl, 1.8 CaCl_2_, 1 MgCl_2_, 10 HEPES, and 5 glucose, adjusted to pH 7.3 using TEAOH for Nav1.5 channels; 137 NaCl, 4 KCl, 1.8 CaCl_2_, 1 MgCl_2_, 10 HEPES, and 5 glucose, adjusted to pH 7.35 using NaOH for KCNQ, Kv4.3, Kv1.5 and Kir2.1 channels. Intercellular (patch pipette) solution contained (in mmol/L): 140 CsAspartate, 2 MgCl_2_, 10 EGTA, 10 HEPES, and 5 MgATP, adjusted to pH 7.2 using CsOH for Cav1.2 channels; 120 CsF, 15 NaCl, 10 EGTA, and 10 HEPES, adjusted to pH 7.2 using CsOH for Nav1.5 channels; 60 KCl, 70 KF, 10 NaCl, 1 MgCl_2_, 11 EGTA and 10 HEPES, adjusted to pH 7.35 using KOH for HCN4, KCNQ, Kv4.3, Kv1.5 and Kir2.1 channels. The amplitudes of hydroxyzine effect on channel activity were compared to vehicle effect in pre-treatment and to positive controls in each cell line.

1. Hamill OP, Marty A, Neher E, et al. Improved patch-clamp techniques for high-resolution current recording from cells and cell-free membrane patches. Pflügers Archiv 1981;391:85-100.

**Supplemental Table 1. Most reported risk factors among the 15 cases in elderly patients**

| **Risk factors of acquired long QT syndrome** | **Cases, n (%)** |
| --- | --- |
| Cardiovascular disorders + concomitant drugs known to induce QT prolongation/TdP | 8 (53.0) |
| Concomitant drugs known to induce QT prolongation/TdP | 3 (20.0) |
| Metabolic abnormalities | 1 (6.75) |
| Metabolic abnormalities + concomitant drugs known to induce QT prolongation/TdP | 1 (6.75) |
| Cardiovascular disorders + renal disease | 1 (6.75) |
| Overdose | 1 (6.75) |
| **Total** | **15 (100)** |

TdP: torsade de pointes; QT: QT interval is defined as the time between the beginning of the Q wave and the end of the T wave of the PQRST cardiac activity cycle.

**Supplemental Table 2. Most reported risk factors among the 39 cases in female patients**

| **Risk factors of acquired long QT syndrome** | **Cases, n (%)** |
| --- | --- |
| Cardiovascular disorders + concomitant drugs known to induce QT prolongation/TdP | 13 (33.5) |
| Overdose + other risk factors | 9 (23.0) |
| Concomitant Drugs known to induce QT prolongation/TdP | 6 (15.5) |
| Overdose | 4 (10.5) |
| Metabolic abnormalities + concomitant drugs known to induce QT prolongation/TdP | 2 (5.0) |
| Cardiovascular disorders | 1 (2.5) |
| Genetic disorders | 1 (2.5) |
| Cardiovascular disorders + metabolic abnormalities + renal disorders | 1 (2.5) |
| Endocrine disorders + concomitant drugs known to induce  QT prolongation/TdP | 1 (2.5) |
| Underlying lupus erythematosus + Polypharmacy | 1 (2.5) |
| **Total** | **39 (100)** |

TdP: torsade de pointes; QT: QT interval is defined as the time between the beginning of the Q wave and the end of the T wave of the PQRST cardiac activity cycle.
